# Being Positive about Selection

**DOI:** 10.1371/journal.pbio.0040087

**Published:** 2006-03-14

**Authors:** Catriona MacCallum, Emma Hill

## Abstract

Detecting positive selection at the molecular level has recently become easier and editors of PLoS Biology face the challenge of identifying standards of excellence in the face of burgeoning interest in this field.

How has language developed in humans and what genetic changes underlie our unique cognitive abilities? Accounts of positive selection that lead to such abilities in humans fascinate us because of the insight they provide into our own evolution, and into the many genetic differences that distinguish us from other apes. The genes that became fixed in our lineage as a result of positive selection are, after all, the ones that make us human. But understanding which gene, or what proportion of a genome, is being driven to fixation by natural selection is of more fundamental biological importance because it can tell us about speciation and the very nature of adaptation.

Relatively recent advances in genomic sequencing and analysis tools have resulted in an explosion of papers on this topic. And as editors of a journal aiming to publish major advances in a field, we face the challenge of identifying standards of excellence in the face of this increasing interest. But the papers vary across many dimensions: they are based on different types of data in a variety of systems and taxa, they use increasingly sophisticated methods, and they address different questions—from targeting disease to understanding the nature of selection and reproductive isolation. Recognizing an advance in this rapidly changing field, where the quality and availability of data also differ substantially, is like trying to catch a moving target.

## Detecting Selection

**Figure 1:**
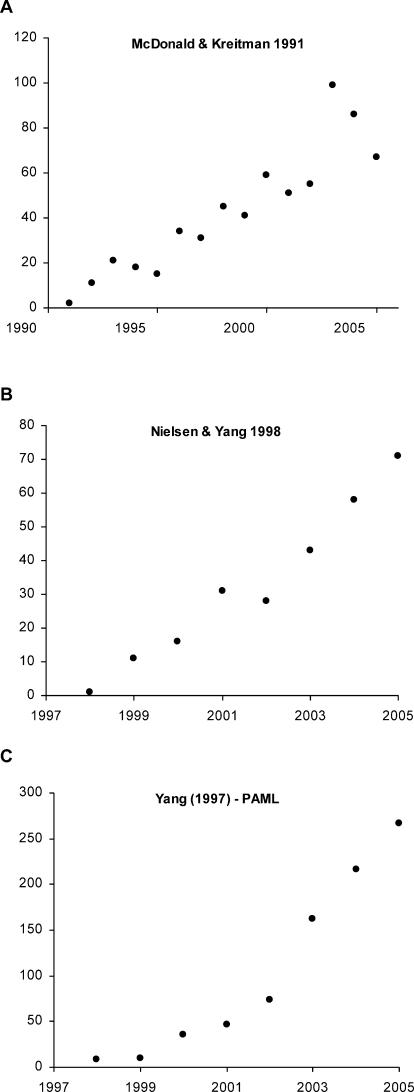
The Increasing Interest in Articles Detecting Positive Selection, as Reflected in the Number of Citations (*y*-Axis) to Some Key Methodological Papers, from Their Publication until the End of 2005 (A) McDonald and Keitman (1991) [1], (B) Nielsen and Yang (1998) [3], and (C) Yang (1997) [4]. Please note that not all articles citing these papers focus only on detecting positive selection. For example, citations to Yang (1997) [4] are to PAML, a computer program that implements many methods in addition to calculating *K*_a_ */K*_s_ ratios. Data collated from ISI Web of Knowledge (Science Citation Expanded) on 17 January 2006.

Most genomic regions are thought to be evolving neutrally; that is, they accumulate mutations (by random genetic drift) that do not influence the fitness of the organism. The traditional measure of whether a protein-coding gene deviates from this and is under positive selection is the relative rate at which nonsynonymous (amino acid–changing) and synonymous (silent) mutations are fixed in a population [1,2]—the *K*_a_ */K*_s_ ratio. If the latter is greater than the former (i.e., *K*_a_ */K*_s_ >1), the assumption is that the gene is changing at a rate faster than would be expected under the neutral theory, and is therefore subject to Darwinian selection. Such a test on whole proteins, however, detects selection in only the more extreme cases. Recently, more powerful methods have focused on detecting selection at the level of individual codons (e.g., [3]), and there are established computer programs, such as PAML [4], that can be used to compare the same gene—codon by codon—in multiple species to pinpoint potential sites of interest. But the wealth of sequence data now available (at least for humans and other model organisms) has meant that positive selection has become almost too easy to detect. Publication of these types of articles is increasing, and there is little sign that interest in this topic is waning (Figure 1).

It is clear, however, that evidence of an excess of amino acid substitutions (at least from site-by-site tests) is no longer a sufficiently convincing demonstration of selection, not only because a high ratio could result from selection on synonymous mutations rather than positive selection on proteins [5], but also because there is potentially a high false discovery rate of selected sites [6]. As one reviewer pointed out soon after we launched *PLoS Biology*, researchers now need to go beyond simply applying canned approaches to detecting positive selection.

## Single-Gene Studies

It is, therefore, no longer appropriate to sequence a gene in several species, stake a claim for positive selection, and expect the results to be published in a top-tier journal. This type of single-gene analysis needs to be augmented by robust experimental evidence for the molecular or functional basis upon which selection would plausibly operate. For example, by combining an analysis of sequence data with a biochemical assay of recombinant proteins, Zhang et al. [7] revealed how positive selection and relaxation of purifying selection shaped the functional divergence of duplicated genes of a digestive enzyme (RNase) in colobine monkeys—and they could attribute the selective force to an earlier change in diet. Of course, estimating the resulting survival and fertility effects of a selected phenotype would provide the most direct evidence of natural selection at the organismal level. In humans at least, such analyses will inevitably be rare, although not impossible, given the availability of some exceptional datasets, such as the Icelandic deCODE database of human pedigrees (e.g., [8]). In addition, the increase in sequence data means that individual genes should not be viewed in isolation, but should be placed in the context of genome-wide patterns to assess whether the signal for selection at an individual locus really differs from the background signal across the genome as a whole.

## Genome-Wide Analyses

Genome-wide analyses of hundreds or thousands of genes can also be used to pinpoint candidate genes or suites of genes. Although one cannot reasonably expect researchers to perform follow-up experiments on every gene, this type of analysis should nonetheless form a starting hypothesis for additional investigation. A common approach is to sort genes by gene ontology (GO) [9] category and speculate on their likely function, involvement in potential pathways, and reasons for being under selection. However, if the purpose of such a scan is to identify candidate genes, then—as with single-gene studies—more detailed follow-up and functional validation of at least some of the key genes of interest are necessary to shore up the evidence. This has to be more than sequencing additional samples and applying further population genetic tests; some kind of experimental data is necessary, from biochemical assays, resolution of structures, cell lines, model organisms, etc., that sheds light on the phenotype being selected.

Genome-wide analyses can inform us about differences between species and the nature of selection more generally. Such studies become interesting when the differences are large or the results unexpected. Independent analyses published in 2002 [10,11] estimated that as much as 50% of the amino acid substitutions between pairs of Drosophila species are under positive selection, a surprisingly large difference given that the bulk of molecular evolution is assumed to be neutral. Subsequently, using an extension of the McDonald–Kreitman test, Andolfatto revealed that a large fraction of the noncoding DNA in these species is also potentially functionally important [12]. Understanding the relative importance of mutation and selection, and to what extent the neutral theory is right or wrong, will remain a key question.

## Give Us the Tools, and We Will Finish the Job

Winston Churchill was right of course; an important factor affecting the rate at which any field advances is the development of new state-of-the-art methods and analyses. And theory, modeling, and database crunching are going to continue to become more important because evolutionary genetic investigators who work on humans or model systems tend not to be limited by data. Indeed, the rise in papers claiming evidence of positive selection has been accompanied by an increase of papers on different methods for its detection. The more innovative studies (e.g., [13,14]) try to tackle the many potential confounding factors clouding the signal of selection, such as demographic effects (i.e., changes in population size and the influence of migration). Others have proposed completely new approaches, which aim to detect different signatures of positive selection, such as selective sweeps, which look for regions of reduced diversity (e.g., [15]). This type of approach has been extended recently to distinguish more recent events from ancient events that have already gone to fixation. For example, Sabeti et al. used the relationship between haplotype frequency and the extent of linkage disequilibrium associated with haplotypes to determine both if and when positive selection might have occurred [16]. And in this issue of *PLoS Biology*, Pritchard and colleagues present their new method (an extension to that of Sabeti et al.) and its application to the Phase I HapMap data to identify human variants under directional selection that have not yet reached fixation [17].

Ideally, such methods papers should provide new insight into how we think about the signature of selection. Moreover, it will be essential that the utility of any new approach is evaluated in comparison with existing methods, and, ideally, that the authors provide a publicly available computer program (as the success of PAML has indicated) to implement the method.

## Prospects

Applying such broad criteria to any paper is a blunt tool for an editor, and it is counterproductive to be too prescriptive. There is no simple “formula” for what makes a good paper on any subject, be it one on a new genome sequence or one exploring sequence evolution. In general, for journals like *PLoS Biology*, papers need to be able to do at least one of the following: significantly address an important general question, present a highly creative and potentially useful approach to a significant problem (and convincingly demonstrate its validity and utility), or ask a completely new yet important broad question and present compelling data bearing on that question.

However, inevitably, a paper on positive selection may stand out, not because of the innovative method used or the extent of the functional follow-up, but because the gene concerned is of particular relevance to our own evolution or has important clinical implications. A classic example of this is the demonstration that the *FoxP2* gene, which may be involved in language acquisition, has been a recent target of selection in the human lineage [18]. Indeed, papers showing what makes humans different will automatically generate a great deal of interest (e.g., most recently [19,20]). Novel twists to positive selection are also intriguing, such as the paper by Zhang and colleagues in this issue of *PLoS Biology* [21], which makes the case for directional selection of a pseudogene in humans—the loss of function of this gene confers a positive advantage in the form of resistance to sepsis.

As editors, we also need to be canny about setting standards for the journal on the basis of one branch of the field with access to one type of data. The data that are now so readily available to human geneticists are not as easily accessed by most evolutionary biologists. There are several groups doing comparable analyses in other model organisms, for example in the fruitfly and in maize, but only one or two outstanding papers have been published so far (e.g., [14,22]), and there are even fewer studies on nonmodel organisms. These data are much harder to acquire and evaluate. Moreover, because there is potentially more scope for follow-up, such species may ultimately provide answers to the more interesting questions, even with relatively less data.

The field is moving fast. An editorial on this topic five years ago would have been very different from the one we write today, and our criteria for publication will no doubt change substantially over the next year. The progress being made is a credit to researchers in the field, and we look forward to constantly reevaluating our editorial standards as new breakthroughs render the most extraordinary contemporary breakthroughs simply ordinary.
